# Dangers from Necrosed Teeth

**Published:** 1890-10

**Authors:** 


ARTICLE VII.

### DANGERS FROM NECROSED TEETH.

Mr. A—— has been under my immediate observation for the past ten years and his case illustrates very forcibly at least one of the dangers of retaining necrosed teeth in their sockets. About nine years age the second bicuspid of the right upper jaw contained a cavity which was filled with amalgam and shortly afterward commenced to ache severely. He insisted on having the filling removed and the nerve killed, which was done. A few months later the tooth died and blackened and a sinus opened through the gingival mucous membrane in the region of the tooth's root. This state of affairs lasted about two years when a series of small abscesses occurred on the right pinna which lasted for about one month and numbered ten or twelve in all. He recovered from the attack and was tolerably comfortable for a few months when the same experience was gone through with, but this seizure was most prolonged and severe. There were marked constitutional symptoms and a large abscess made its appearance in the right temporal region. These attacks returned at intervals of a few weeks or months for several years, each one being slightly more severe than the last and finally involved the scalp of the right side. About this time a large abscess made its appearance on the knuckle of the little finger of the left hand, and the man commenced to show signs of marked reduction of health and strength. I have inquired of several dentists as to whether they had ever known of a similar case and they invariably answered in the negative. I further notice that the vent in the mucous membrane closed and it was shortly after such closure that the abscesses made their appearance. At last I decided to have the tooth extracted, which was done nearly a year ago, and since that time the patient has been in perfect health which was an experience he had not known nearly all the time the dead tooth was in his head.

The root of the tooth was very much softened and roughened, while the tooth-socket and tissues surrounding it were quite sensitive to pressure. To my mind, this was a case of septicœmia, which I can readily conceive to be the result of pent-up pus in the neighborhood of the dead root. I would earnestly recommend the removal of all dead teeth, for while we all know that necrosed teeth may be retained indefinitely without causing trouble in many instances, on the other hand one such case as the above is sufficient to demonstrate that there is always impending danger of either septicœmia, pus in the antrum of Highmore, necrosis of the alocolus, or numerous kindred affections while a dead tooth is left in the jaw.—*William P. Beach, M. D., in the Brooklyn Medical Journal.*

The above is extremely interesting as long as the author confines himself to a history of the case. When he advises the extraction of all "dead" teeth we must ask what he intends the expression "dead" to signify. If he means all teeth in which what he calls "the nerve" has been killed we must take issue with him. Pulpless teeth may and frequently should be retained. In a large majority of such cases it would be malpractice to extract. If, however, we may understand by "dead teeth" those where after the loss of the pulp, disease has destroyed the vitality of the pericementum, we say most peremptorily that they should be extracted. There should be no exception to this rule. Such a tooth is an offense which nature will not tolerate, and the effort to be rid of the intruder results in the formation of pus which, either absorbed or swallowed, may cause septicœmia as above related.

Dr. Atkinson recently reported a case in which septicœmia traceable to the teeth resulted in abscesses on the finger and yet another where abscesses appeared on the femur, and great toe.—*Dental Mirror.*

